# Core Shell Nanostructure: Impregnated Activated Carbon as Adsorbent for Hydrogen Sulfide Adsorption

**DOI:** 10.3390/molecules27031145

**Published:** 2022-02-08

**Authors:** Nurul Noramelya Zulkefli, Rajeevelosana Seladorai, Mohd Shahbudin Masdar, Nabilah Mohd Sofian, Wan Nor Roslam Wan Isahak

**Affiliations:** 1 Department of Chemical & Process Engineering, Faculty of Engineering & Built Environment, Universiti Kebangsaan Malaysia, UKM, Bangi 43600, Selangor, Malaysia; p97653@siswa.ukm.edu.my (N.N.Z.); A158273@siswa.ukm.edu.my (R.S.); wannorroslam@ukm.edu.my (W.N.R.W.I.); 2 Fuel Cell Institute, Universiti Kebangsaan Malaysia, UKM, Bangi 43600, Selangor, Malaysia; nabilah@ukm.edu.my; 3 Research Centre for Sustainable Process Technology (CESPRO), Faculty of Engineering & Built Environment, Universiti Kebangsaan Malaysia, UKM, Bangi 43600, Selangor, Malaysia

**Keywords:** biogas purification, adsorption, core shell, impregnated activated carbon, nanostructure

## Abstract

This study focuses on the synthesis, characterization, and evaluation of the performance of core shell nanostructure adsorbent for hydrogen sulfide (H_2_S) capture. Commercial coconut shell activated carbon (CAC) and commercial mixed gas of 5000 ppm H_2_S balanced N_2_ were used. With different preparation techniques, the CAC was modified by core shell impregnation with zinc oxide (ZnO), titanium oxide (TiO_2_), potassium hydroxide (KOH), and zinc acetate (ZnAC_2_). The core structure was prepared with CAC impregnated by single chemical and double chemical labelled with ZnAC_2_-CAC (single chemical), ZnAC_2_/KOH-CAC, ZnAC_2_/ZnO-CAC, and ZnAC_2_/TiO_2_-CAC. Then, the prepared core was layered either with KOH, TiO_2_, NH_3_, or TEOS for the shell. The synthesized adsorbents were characterized in physical and chemical characterization through scanning electron microscopy (SEM), thermal gravimetric analysis (TGA), and Brunauer-Emmett-Teller (BET) analyzers. Operation of the adsorber column takes place at ambient temperature, with absolute pressure at 1.5 bar. The H_2_S gas was fed into the column at 5.5 L/min and the loaded adsorbents were 150 g. The performance of synthesized adsorbent was analyzed through the adsorbent’s capability in capturing H_2_S gas. Based on the results, ZnAc_2_/ZnO/CAC_WOS shows a better adsorption capacity with 1.17 mg H_2_S/g and a 53% increment compared to raw CAC. However, the degradation of the adsorbents was higher compared to ZnAc_2_/ZnO/CAC_OS and to ZnAc_2_/ZnO/CAC_WS ZnAc_2_/ZnO/CAC_OS. The presence of silica as a shell has potentially increased the adsorbent’s stability in several cycles of adsorption-desorption.

## 1. Introduction

Biogas is produced from the decomposition of organic waste using microorganisms without the presence of oxygen. The anaerobic process degrades organic matter through microorganisms, prior to the formation of biogas without oxygen. The biogas production from anaerobic processes produces up to 40–75% of methane (CH_4_), 25–40% of carbon dioxide (CO_2_), 0.5–2.5% of nitrogen (N_2_), 10–30 ppm(v) of ammonia (NH_3_), and 1000–3000 ppm(v) of hydrogen sulfide (H_2_S) [1,2,3]. However, the concentration of product in biogas depends on the source of fed substrate, temperature pressure for the biogas process. The early-stage elimination of H_2_S gas was recommended for biogas purification to prevent any causes of degradation in biogas production. This is because biogas has great potential for future innovation and as an alternative energy source. Energy produced from purified biogas can be used as cooking fuel and for generating electricity [4]. Moreover, purified biogas is used to run combustion engines with better performance compared to raw biogas [5].

In industrial applications, H_2_S removal can be carried out using several methods, namely the Claus process, biological, absorption, membrane unit, and adsorption [3,6,7,8]. The adsorption process is the most effective method for the removal of H_2_S by using adsorbents such as raw activated carbon (CAC), single impregnated CAC, dual impregnated CAC, and core shell. The adsorption process is divided into chemical adsorption and physical adsorption. Physical adsorption of gases occurs when the attraction between the solid and gas adsorption molecules is greater than that between the gas molecules themselves. Chemical adsorption is the transfer or sharing of electrons, or their break down into atoms or radicals that are bound separately. Moreover, the adsorption technique through activated carbon requires optimal conditions to function efficiently.

The adsorption process is the most effective method for the removal of H_2_S with low concentration by using adsorbents via mesoporous materials. The mesoporous materials used in this study were activated carbon type, which is known to have a high surface area, microspores, thermal stability, a high removal capacity, and a low cost per unit volume. On the other hand, the performance of CAC was low in terms of adsorption capacity. In order to enhance the adsorption capability, the activated carbon needs to modify the adsorbents surface through chemical impregnation. As H_2_S is selective toward certain chemicals such as iron oxide (FeO), iron hydroxide (FeOH), and zinc oxide (ZnO) [9], several chemicals were selected based on the suggestions of other researchers.

Homogeneous distribution of the adsorption agent on the internal surface of the CAC is important. In addition, microspore and macrospore barriers should be avoided to maintain a bonding agent on the surface of the reaction material. Therefore, core shell is one of the methods to increase particle stability and dispersibility of the core particle and the surface can be modified [10]. A significant amount of nanomaterial atom is on the surface, which increases chemical activity in nanomaterials [11]. According to the study [12], core-shell structure H_2_S imprinted polymers exhibited high adsorption capacity at ambient temperature with the presence of water and steam, and they are very efficient for H_2_S and CO_2_ separation. Solid core and porous shell provide a higher surface area.

Core shell silica material has been widely used for highly efficient separation with a high flow rate and low back pressure. The core shell contains nano particles and includes one or more dimensions in the nanometer scale (100 nm). Synthesis, characterization, and application of core shell or nanostructured core are among the most important parts of various nanotechnologies [13]. Small particle size increases the surface area to volume ratio. However, synthesis of core shell is a complex process and there are a variety of techniques for producing different types of cores. The nanostructure and the surface of the core shell have many advantages, namely electrical conductivity and dielectric properties.

Therefore, in this study, the CAC were synthesized through core shell methods and the performance of adsorbents was investigated through adsorption capability of H_2_S by using the commercial mixed gas of 5000 ppm H_2_S balanced N_2_ to mimic the concentration of H_2_S gas in most biogas production. The physical and chemical characterizations of these adsorbents were determined using SEM-EDX, BET, and TGA.

## 2. Materials and Methods

### 2.1. Materials

**Figure 1:**
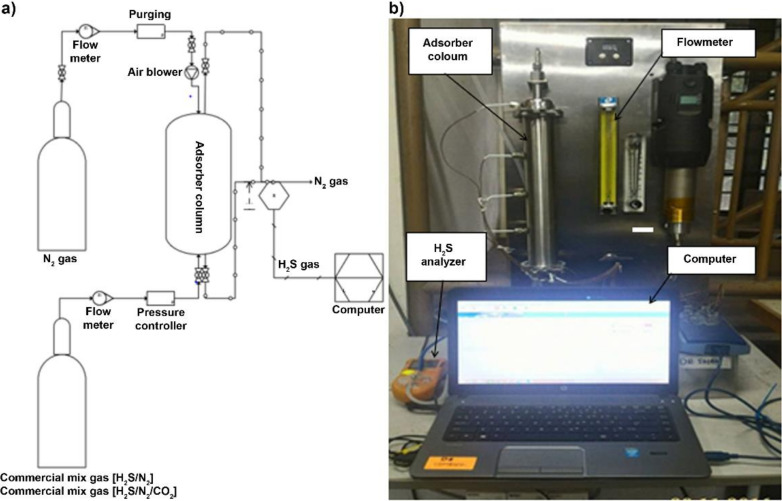
Adsorber column. (**a**) Schematic diagram of operating adsorber column. (**b**) Actual photo of experimental setup.

The granular commercial coconut activated carbon (CAC) was purchased through Effigen Carbon Shd. Bhd. Kapar, Selangor, Malaysia. The CAC was sieved to obtain a particle size in the range of 3–5 mm. The selected CAC impregnation compound was zinc acetate (ZnC_4_H_6_O_4_), potassium hydroxide (KOH), titanium oxide (TiO_2_), 95% ammonia (NH_3_), and tetraethyl orthosilicate (TEOS), which were purchased from Friendemann Schmidt Chemicals, Malaysia and used as obtained without prior purification. Then, the laboratory-scale experiments were conducted in a continuous atmospheric packed bed reactor, or adsorber column. The laboratory-scale experimental setup diagram is presented in Figure 1. The inner diameter of the adsorber column was 0.5 cm, bed length was up to 10 cm, and total column volume was 0.2 L. The commercial mixed gas of 5000 ppm H_2_S balanced N_2_ was obtained from Linde Malaysia Sdn Bhd and it was used as feed gas throughout this study. As mentioned earlier, H_2_S production in the biogas system was in low concentration, at approximately 0.1 to 1%. Previously, the measurement of gas composition was implemented for several samples of biogas from palm oil mill at which the concentration was approximately 3000 to 5000 ppm. Therefore, the maximum concentration for H_2_S of 5000 ppm was chosen to represent the H_2_S concentration in the biogas. Synthetic biogas was not used in this study because the intention of this study was to focus on the potential of adsorbents for H_2_S gas using a similar feed gas with different synthesized adsorbents.

### 2.2. Preparation of Core Shell Activated Carbon (CS/CAC)

**Figure 2:**
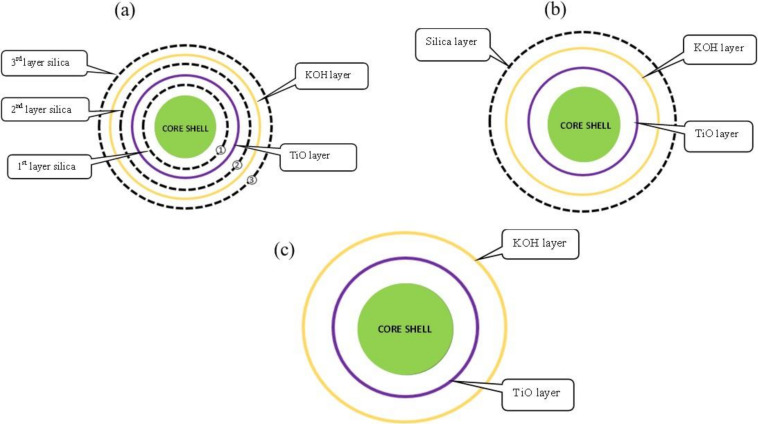
Core shell structures. (**a**) Core shell with multiple silica layers. (**b**) Core shell with single layer silica. (**c**) Core shell without silica layer.

As in the study by Zulkefli et al. [9,14], the 350 g of CAC was impregnated first with 0.2 M of chemical impregnation on the CAC surface as core preparation. After the core was prepared, the shell method was prepared with 0.2 M solution of each chemical compound (KOH, TiO_2_, NH_3_ and TEOS), with 600 mL of distilled water in a beaker which was heated on a hot plate up to 65 °C. The prepared core was soaked into the solution, and it was prepared for 30 min at each shell process. Next, the solution was filtered, washed several times, and then dried at 120 °C for 24 h. All the adsorbents prepared for core and shell with silica at each shell layer, silica at outer shell, and no silica were used as CS/CAC_WS, CS/CAC_OS, and CS/CAC_WOS, as shown in Figure 2.

### 2.3. Characterization of Adsorbents

All the synthesized adsorbents were undergoing several physical and chemical characteristics such as Brunauer-Emmett-Teller (BET), scanning electron microscopy (SEM), and thermal gravimetric analysis (TGA). The surface morphology and chemical composition of DCM adsorbent particles were investigated using a scanning electron microscope (EDAX APOLLO X model, Mahwah, NJ, USA) and a CARL ZEISS EVO MA10 (Jena, Germany), respectively. Under an accelerating voltage of 10 kV, the analysis was carried out in order to visualize the details of adsorbent properties in terms of structural particles and weight percent elements present on the surfaces of the adsorbents, among other things. A further investigation into the properties of the adsorbents was carried out using the BET calculation method, which was performed using Micrometric ASAP 2010 Version 4.0 (Micrometric, Lincoln, UK). N_2_ adsorption-desorption isotherms were used to determine the specific surface area and porosity of the soil sample. The thermal stability of the adsorbents was then determined using the TGA method (TGA-50, Shimadzu, Kyoto, Japan). According to the study, weight loss was observed as a result of the temperature change. The amount of 1 g of granular DCM adsorbents was heated to 600 °C at a rate of 10 °C/min under a flow rate of 20 mL/min of purified air at a temperature of 10 °C/min.

### 2.4. Operation of H_2_S Adsorber Column

In a lab-scale experimental apparatus based on a fixed-bed reactor, as previously described by Zulkefli et al. [9,14], H_2_S dynamic adsorption tests were carried out at T = 30 °C and total P = 1 atm in a fixed-bed reactor. A total of 150 g of DCM adsorbent was loaded into a stainless steel fixed-bed column (D_in_ = 0.06 m) to conduct the separation. In order to maintain a constant feed flow rate of 5.5 L/min for each run, the commercial mixed gas of H_2_S/N_2_ (5000 ppm H_2_S balanced N_2_) was used as feed.

As a result of the tolerable range for gas exposed to the environment and to fuel cell devices [9], the H_2_S breakthrough gas concentration at the outlet stream was fixed at 5–10 parts per million (ppm). Testing for H_2_S concentrations was carried out using a custom-built portable H_2_S analyzer (model GC310), which was equipped with an electrochemical selective sensor for H_2_S that operated in the range of 0–1000 parts per million (ppm). The data acquisition and elaboration were carried out by interfacing the analyzer with a PC unit that contained the H_2_S analyzer’s monitoring software already installed. The adsorption capacity of H_2_S gas in CAC was calculated using the following formula [4]:

(1) $$q=\frac{Q\;\times {\;T}_{b}\times {\;C}_{o}\times {\;\mathrm{MW}}_{H_{2}S}}{V_{m}\times {\;M}_{\mathrm{Ads}}}$$

where,

q = Capacity of adsorption (mg H_2_S/g adsorbent)

Q = Flow rate of H_2_S gas (L/min)

T_b_ = Breakthrough time (min)

C_o_ = Breakthrough concentration (kg/L)

MW_H2S_ = Molecular weight of H_2_S (kg/kmol)

V_m_ = Molar volume (L)

M_Ads_ = Adsorbent weight (g)

Following that, the desorption process for each of the adsorbents was carried out in the same circumstance as the set-up preparation in the previous study by Zulkefli et al. [9,14]. Adsorbents that have been saturated with H_2_S go through a three-step purging process. In the first step, the spent adsorbents are run with an air blower for 30 min at 150 °C with a flow rate of 100 L/min. Second, the same operating parameters were applied to the column for 30 min without the use of a temperature controller. The final stage involved the introduction of N_2_ gas into the stream, which was fed at a rate of 5.5 L/min for 30 min in order to purge and stabilize the surface adsorbents before they were ready to be used for the next adsorption procedure.

## 3. Results and Discussion

### 3.1. H_2_S Adsorption Capacity

#### 3.1.1. The Performance of CS/CAC_WS

Silica-based terraces take into account the chemical compounds used in several layers involving TEOS-type silica for each CS/CAC layer. According to the study of Elyassi et al. [15], the combination of silica and activated carbon elements also performed well as H_2_S gas absorber material. The casing core is a type of nanoparticle consisting of core (internal material) and casing (outer coating material) [13]. Silica is chosen as a coating material because it has several advantages over other organic matter; it can reduce bulk conductivity and improve the stability of the suspended material for core parts particles. In addition, silica is a very inert chemical as it can be located on the core surface without interrupting the redox reaction on the surface [13]. Although silica has the advantage of being a coating material compared to other chemicals, the advantages of adding silica layers to the CAC core are considered as hydrogen sulfide gas adsorbents.

**Figure 3:**
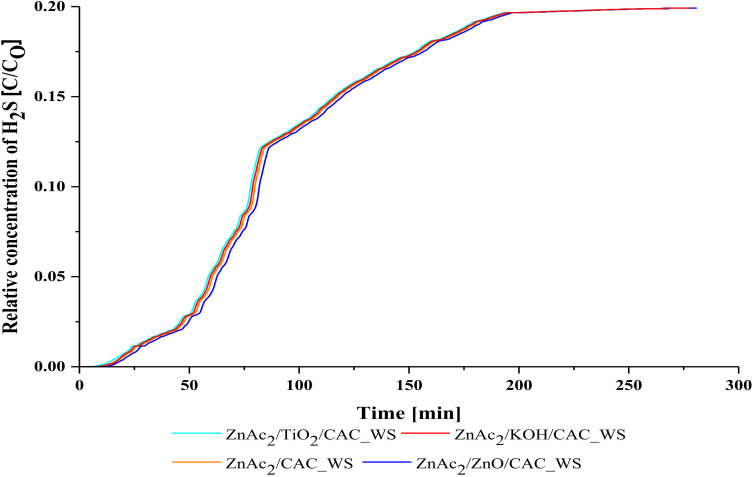
Relative concentration of H_2_S profile for CS/CAC_WS adsorbents.

The adsorption performance of the ZnAc_2_/CAC_WS, ZnAc_2_/ZnO/CAC_WS, ZnAc_2_/TiO_2_/CAC_WS, and ZnAc_2_/KOH/CAC_WS adsorbent materials is shown in Table 1 and Figure 3 below. ZnAc_2_/ZnO/CAC_WS absorber material has H_2_S gas absorption for CS/CAC_WS adsorbent material development. Compared to the adsorption capacity obtained in the development of CS/CAC type adsorbents throughout the selection of single impregnation core (ZnAc/CAC_WS) was seen as a decline compared to the core of dual chemical (DCM). This may be due to an increase in active sites on the adsorbents surface that enhanced the capability of adsorption of ZnAc_2_/ZnO/CAC_WS adsorbents [14].

Thus, the adsorption performance of CS/CAC_WS adsorbents with a fed concentration of 5000 ppm H_2_S shows significant effect toward selection of core, which adsorption was slightly higher in DCM/CAC as a core compared to SI/CAC. Hence, the ZnAc_2_/ZnO/CAC_WS adsorbent showed higher adsorption capacity compared to other adsorbents. This improvement is a benchmark for the development performance of this absorbent material to adsorb gas optimally.

#### 3.1.2. The Performance of CS/CAC_OS

**Figure 4:**
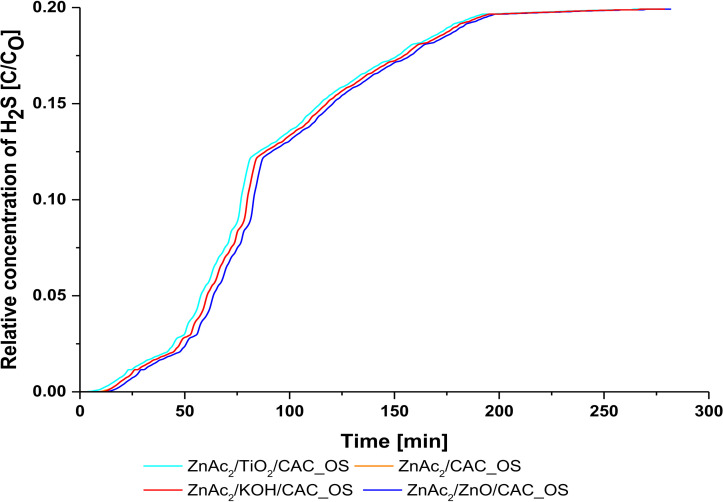
Relative concentration of H_2_S profile for CS/CAC_WS adsorbents.

Subsequently, the CS/CAC method for silica (TEOS) use in the outer layer was developed. Four (4) types of adsorbents were developed based on the performance of the adsorption materials in the SI/CAC and DCM/CAC methods used as the core of this study. Several compounds form layers as a clone to this core. Then, the last layer uses TEOS compounds as silica in the development of CS/CAC_OS adsorbents. Table 2 and Figure 4 show the performance of the adsorption capacity for each adsorbent.

The ZnAc_2_/ZnO/CAC_OS adsorbents showed optimum performance with H_2_S adsorption capacity at 0.67 mg H_2_S/g compared to ZnAc_2_/TiO_2_/CAC_OS (0.54 mg H_2_S/g) > ZnAc_2_/KOH/CAC_OS (0.50 mg H_2_S/g > ZnAC/CAC_OS. Additionally, the preparation of ZnAC2/ZnO/CAC_OS adsorbent showed an improvement in the performance of H_2_S adsorption capacity compared to the development of ZnAc2/ZnO/CAC_WS adsorbent material up to 19.4%. Thus, the use of one layer of TEOS on the outer surface of this slider improves the performance of H_2_S gas adsorption better than the use of TEOS to three layers as in the development of CS/CAC_WS adsorbents.

#### 3.1.3. The Performance of CS/CAC_WOS

**Figure 5:**
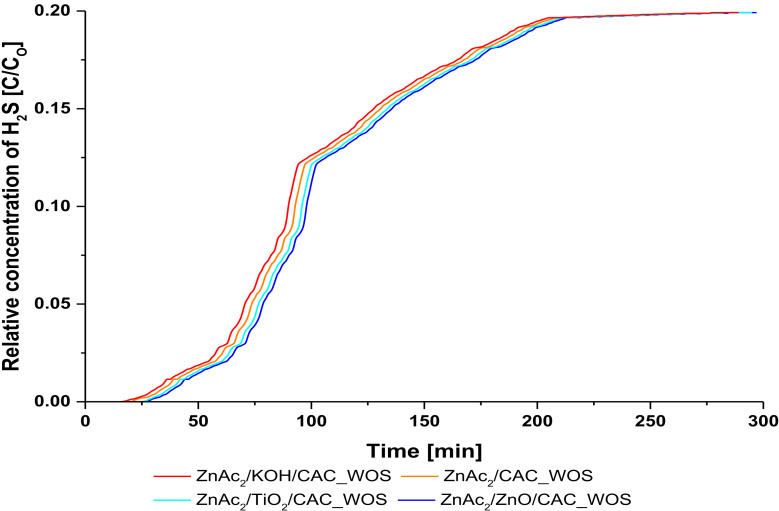
Relative concentration of H_2_S profile for CS/CAC_WOS adsorbents.

The development of CS/CAC_WOS adsorbents demonstrate the performance of different H_2_S adsorption capacity with the development of CS/CAC_WS and CS/CAC_OS adsorbents where the performance of these adsorbents is identified to have a more positive impact on the adsorption of H2S gas. Preparation of CS/CAC_WOS adsorbent material developed with two main layers of compounds KOH and TiO_2_, which represent a slit to the core without any coating from TEOS, have been better able to adsorb H_2_S gas. The performance of this absorbent material is recorded as shown in Table 3 and Figure 5.

The adsorbent material with the ZnAc_2_/ZnO/CAC core performs well compared to other core uses. Development of this ZnAc_2_/ZnO/CAC_WOS adsorbent showed the optimum H_2_S gas adsorption capacity at 1.17 mg H_2_S/g compared to ZnAc_2_/TiO_2_/CAC_WOS, ZnAc_2_/CAC_WOS and ZnAc_2_/KOH/CAC_WOS. However, the performance of the ZnAc_2_/KOH/CAC_WOS adsorption capacity shows a different pattern of adsorption than the ZnAc_2_/KOH/CAC_WS and the ZnAc_2_/KOH/CAC_OS adsorbents which have lower performance compared to the adsorbent material of the ZnAc_2_/CAC core. This is likely due to the high blockage of KOH compounds to the surface of the adsorption, which is the active surface. In conclusion, the ZnAc_2_/ZnO/CAC_WOS adsorbent material showed an increase of 42.7% and 53.9% compared to the ZnAc_2_/ZnO/CAC_OS and the ZnAc_2_/ZnO/CAC_WS absorbers.

The performance shown by the core with metal acetate compounds and metal oxides is capable of good H_2_S gas adsorption compared to metal and based compounds. This is because oxide metals, such as zinc oxide (ZnO), have a high efficiency with respect to desulfurization of, e.g., coal gas and hydrogen gas for fuel cells, depending on their acid-bes properties [16]. Moreover, according to Yang et al. (2018), an increase in the number of compounds (voltage rates) can cause gas difficulties to penetrate the adsorbent surface and absorb well, following a thick layer of the clones. Thus, the performance shown by the lower CS/CAC_WS and CS/CAC_OS adsorbents relative to CS/CAC_WOS is due to the thickness of the compound (leaning), which causes the difficulty of the H_2_S gas to penetrate the core surface. Furthermore, based on studies of NOx and SO_2_ gas adsorption on the same type of compound that showed a decrease in gas vapor capacity [17], the selection of compounds for the development of CS/CAC type adsorbents also plays a role in optimizing gas absorption.

Consequently, ZnAc_2_/ZnO/CAC_OS adsorbent material is identified as the best chemical compound composition as it achieves the highest H_2_S adsorption capacity. However, the discussion on this study was followed by a characterization method on each absorbent through BET and TGA analysis to identify surface properties and thermal stability of the developed adsorbent material. Then, the performance of each of these adsorbents was used in the next study activity to identify stability in the regeneration of the adsorbent material by optimizing the parameters for the development of the adsorbent material.

### 3.2. Characterization of Fresh Adsorbents

#### 3.2.1. SEM-EDX Analysis

**Figure 6:**
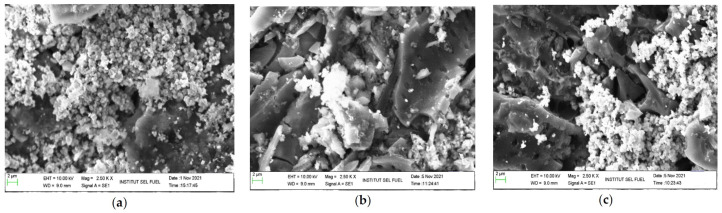
SEM micrograph images for CS/CAC adsorbents at magnification of 2.5 Kx and 2-micron scale: (**a**) ZnAc_2_/ZnO/CAC_WS, (**b**) ZnAc_2_/ZnO/CAC_OS, and (**c**) ZnAc_2_/ZnO/CAC_WOS.

Figure 6 is a micrograph image of SEM for absorbent material provided for CS/CAC type adsorption study with magnification at 2.5 Kx and 2-micron scale. Based on the observations on this micrographic image, the morphological structure shows the presence of blurred white spots that form the coating as a basis for the presence of chemical compounds on the surface of the adsorbent material. However, the presence of elements on the surface of this absorbent material cannot be explained solely by image because this morphological structure does not show quantitative data. Thus, further studies through the EDX method are needed to identify the presence of elements on the surface of this absorbent material.

Table 4 shows the presence of elements on the adsorbents surface for all CS/CAC types, namely CS/CAC_WS, CS/CAC_OS, and CS/CAC_WOS. Element O shows an increase in core preparation with a combination of chemicals (ZnAc_2_/ZnO, ZnAc_2_/TiO, ZnAc_2_/KOH) compared to the single chemical used (ZnAc_2_/CAC). The enhancement of O elements on the surface of the adsorbent material plays an important role in helping to improve the efficiency of H_2_S gas adsorption [18,19]. The ZnAc_2_/ZnO/CAC_WOS adsorbent has the highest mass percentage of element O which proves that this adsorbent material has the best adsorption with the highest H_2_S vapor capacity.

However, the sample selection used for this characterization is random and at values on the morphological structure layer and the presence of elements based on the sample used. Morphological structures do not show quantitative data, and it is difficult to make significant assessments of the presence of elements on the surface of the adsorbent material. The increase in elements such as K, Ti for ZnAc_2_/KOH, and ZnAc_2_/TiO_2_-based adsorbents is due to the addition of these compounds as each CS/CAC adsorbent material is coated with the same compound. This increase is different because the initiation process for the preparation of the core from the same compound has increased compared to the adsorbent material from the ZnAc_2_/ZnO and the ZnAc_2_ as the cores.

In addition, the presence of Si elements can be observed in CS/CAC_WS and CS/CAC_OS adsorbents following the use of TEOS in the preparation of this absorbent material. The percentage of mass obtained through this EDX method found that CS/CAC_WS was highest compared to CS/CAC_OS due to the use of TEOS in each CS/CAC_WS layer preparation process. Meanwhile, CS/CAC_OS absorber material uses only TEOS on the surface of the last layer of the adsorbent material. The high percentage of Si element in CS/CAC_WS has a less favorable effect following H_2_S gas adsorption and it is very low compared to other CS/CAC types.

However, the use of TEOS compounds in CS/CAC_OS can absorb low H_2_S gas compared to other adsorbents such as CS/CAC_WOS and DCM/CAC. Thus, further studies on the stability of adsorbents in the H_2_S gas frequency cycle are needed to determine the capabilities of these CS/CAC absorbers. The preparation of CS/CAC adsorbents has increased the ability to adsorb H_2_S gas through changes in the formulation of new pores on observable CAC surfaces as in the morphological structure in Figure 6. The selection of compounds also plays an important role in helping the efficiency of gas vapor as the adsorption of H_2_S gas is selective.

#### 3.2.2. BET Analysis

**Figure 7:**
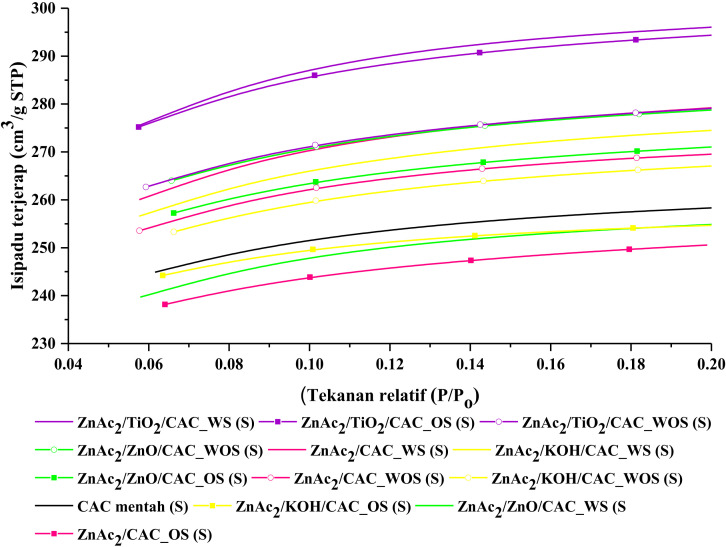
N_2_ adsorption-desorption isotherm.

Characterization of the surface properties of the adsorbent material has been performed on all CS/CAC adsorbents. These surface properties involve the coating on the BET surface area, pore size, and average pore volume for each adsorbent. The determination of surface area and pore size is based on the analysis process of N_2_ gas vapor on each adsorbent. Figure 7 and Table 5 are N_2_ adsorption isotherms of the adsorbent material. Based on Figure 7, the isothermal for all adsorbents is a Type I (B) based on the IUPAC classification [20]. A Type I profile that is curved with a sharp, emphasized relative pressure (P/PO) axis at relatively low pressures to reach the Langmuir isotherm. Generally, the Langmuir isotherm is obtained from the adsorption of monomolecular gas by porous adsorbents. The isothermal of Type I also shows that this adsorbent material is of a type of microbial material with a diameter smaller than <2.5 nm [20,21].

The increase in the volume of N_2_ gas adsorption can be seen to grow faster as adsorption occurs in micropores, and then the vapor curve slowly increases following the adsorption that occurs after the micropores, which is on the outer surface of the adsorbent material. Figure 7 shows the volume of adsorbed N_2_ gas as the following adsorbent ZnAc_2_/TiO_2_/CAC_WOS (S) > ZnAc_2_/ZnO/CAC_WOS (S) > ZnAc_2_/ZnO/CAC_OS (S) > ZnAc_2_/CAC_WOS (S) > ZnACS.

As shown in Table 5, quantitative findings from the analysis of the volume of adsorbed substances show that the BET surface area can affect the adsorption efficiency of the adsorbent material. However, this efficiency does not depend solely on the surface area. As previously obtained, the efficiency of the CS/CAC_WOS adsorbents material shows a higher abrasive efficiency than other adsorbent materials. However, BET surface area for CS/CAC_WOS adsorbents is higher than other adsorbent materials, especially raw CAC. Thus, the pore size of each of these adsorbents also contributes to the adsorption efficiency where CS/CAC_WOS has a larger pore size than other adsorbents, which is about 1.7% higher than raw CAC. CS/CAC_WS adsorbents were also observed to have higher S_BET_s with lower pores than other absorbent materials. This is likely to result in chemical blockage of CS/CAC_WS adsorbent pores. This blockage affects the reduction of active sites aimed at increasing the adsorption of H_2_S gas.

In addition, the high micropore area of this CS/CAC_WOS adsorbent also contributes to effective H_2_S adsorption where it is assumed that H_2_S adsorption is better [22]. This high micropore area provides the probability of forming higher active sites that ultimately help the absorption of adsorbents. Not only that, the difference in surface area and in pore size is likely due to the formation of a compound layer on the microbial surface, which has been proven through previous EDX characterizations.

However, in the case of the development of this CS/CAC adsorbent, this layer or slider acts as a resistance to the penetration of H_2_S gas into each sloping to the core, which can enhance the catalyst oxidation process and gas adsorption rate at each layer of the slit. The use of silica (TEOS) also plays a role in helping to stabilize the adsorption. Thus, this CS/CAC method can enhance H_2_S gas adsorption activity and enable the life of the adsorbent in the process of regeneration.

#### 3.2.3. TGA Analysis

**Figure 8:**
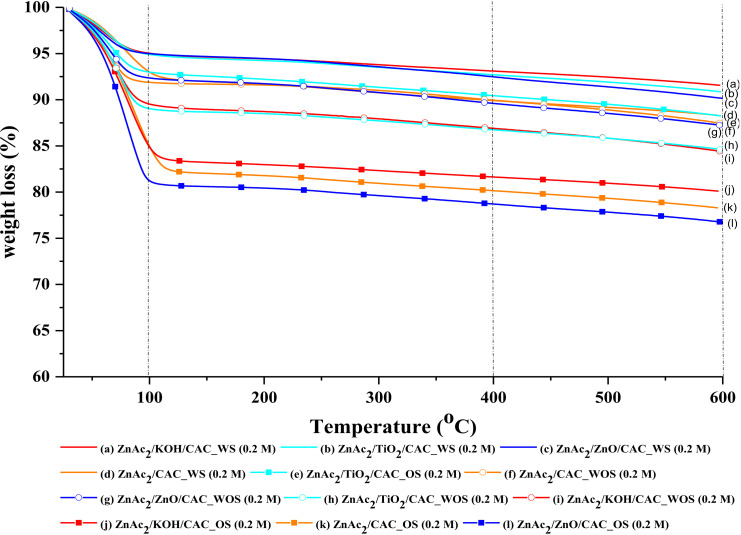
TGA profile for CS/CAC adsorbents.

Characterization through TGA analysis was performed for each selected fresh absorber material. This TGA analysis was conducted between 25 °C to 600 °C to identify the mass reduction of each absorbent material in three phases of temperature derivative. Among the three temperature phases seen are in phases (i) 25–100 °C; (ii) 100–400 °C; and (iii) 400–600 °C. Figure 8 and Table 6 show the following adhesion to the percentage of mass loss on the derivative of the temperature. Generally, the reduction of the mass percentage in the temperature derivative for phase (i) is associated with a reduction in the moisture content of the absorbent material. On average, the moisture loss in phase (i) was about 4.9–15.4%.

Generally, the reduction of the mass percentage in the temperature derivative for phase (i) is associated with a reduction in the moisture content of the absorbent material. On average, the moisture loss in phase (i) was about 4.9–15.4%. However, the presence of minimal moisture helps to improve the adsorption of H_2_S gas and the actual adsorption-desorption results found that CS/CAC adsorbents were better to adsorb H_2_S gas than raw CACs. This can be attributed to the presence of moisture in the adsorbent material where a minimum temperature derivative of phase (i) was <10%, which is capable of increasing the adsorption of H_2_S gas. This loss of moisture is within the normal range as per researchers Vinodhi et al. [23] who found that the percentage of moisture loss was below 20%.

The stability of the adsorbent material is seen in phase (ii) and phase (iii) of the temperature derivative which tests the stability of the adsorbent material against temperature use. The percentage of mass loss in both phases was below 6% for each temperature derivative. Thus, this adsorbent material can be concluded to have stability against high temperature use which is up to 600 °C. The presence of minimum moisture in the adsorbent also affects the adsorption of the adsorbent. The presence of element O and of moisture in the adsorbent material may be factors in the increase of the adsorption of H_2_S gas. This can be attributed to the finding that the presence of high O elements increases the oxidation of the TiO_2_ compounds and facilitates the adsorption of NOx and SO_3_ gases [24,25].

### 3.3. Regeneration of Adsorbents

**Figure 9:**
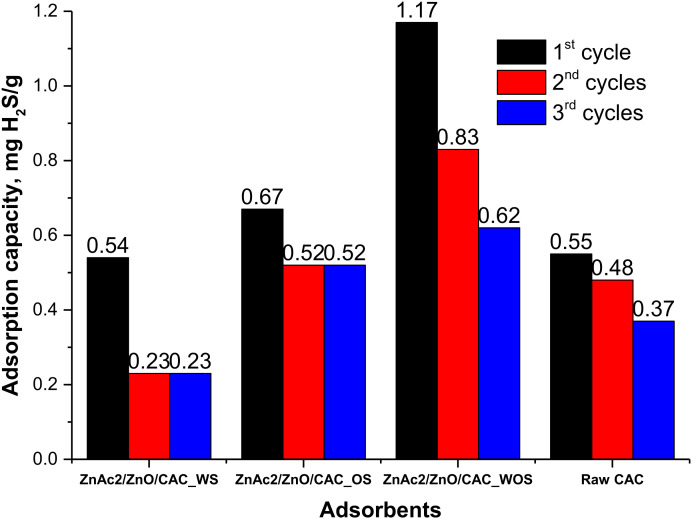
Regeneration performance of adsorbents throughout three adsorption-desorption cycles.

The regeneration of absorbent materials plays an important role in ensuring that the life of the absorbent material is longer. Not only that, the regeneration of these adsorbents also plays an important role in absorbing gas, especially H_2_S gas for several vapor frequency cycles to prevent excessive secondary waste disposal. Commonly, the H_2_S gas adsorption has a detrimental effect on the adsorbent surface which may be difficult to repair or to reuse, following strong S element bonding on the CAC surface. However, the development of various adsorbents was studied to identify the permeability of H_2_S gas frequency and to provide optimum vapor levels for several cycles. Thus, Figure 9 shows a comparison of H_2_S vapor capacity for all adsorbents with 3 cycles of adsorption-desorption. Percentage reduction per cycle can be observed for each CS/CAC_WS adsorbent material.

Each cycle of adsorption-desorption has shown a degradation in the efficiency of the adsorbent. For ZnAc_2_/ZnO/CAC_WS adsorbents at 150 °C, the decrease in the efficiency of the second cycle was 57.4% compared to the first cycle. Then, in the third cycle, the efficiency of the adsorption-desorption remained as in the second cycle. Besides, the degradation in ZnAc_2_/ZnO/CAC_OS showed a 22.4% decrease in the efficiency of the second cycle and it remained present in the third cycle. The ZnAc_2_/ZnO/CAC_WOS had a higher capability in capturing H_2_S compared to ZnAc_2_/ZnO/CAC_WS and to ZnAc_2_/ZnO/CAC_OS. However, in terms of degradation, the second cycle showed a decrease of approximately 29.1% and a further degradation in the third cycle of 25.3%. The stability of adsorbents was slightly weaker than adsorbents with silica. ZnAc_2_/ZnO/CAC_OS is capable of adsorping H_2_S gas 19% better than ZnAc_2_/ZnO/CAC_WS, although the silica quantity also affected the performance of H_2_S adsorption.

## 4. Conclusions

Using different preparation techniques, the CAC was modified and synthesized by core shell impregnation with zinc oxide (ZnO), titanium oxide (TiO2), potassium hydroxide (KOH), and zinc acetate (ZnAC2) for H_2_S capture. The characterization and the adsorption-desorption performance test were implemented for synthesized adsorbents. The core selection influences the capability of adsorbents in H_2_S capture, with the result that dual chemical impregnation (ZnAc_2_/ZnO/CAC_WS) was 39% higher when compared to that of single impregnation (ZnAc_2_/CAC_WS). The ZnAc_2_/ZnO/CAC_WOS results in a better adsorption capacity of 53%, compared to raw CAC. However, the performance of the core shell in adsorbing H_2_S decreases after the first cycle, due to the adsorption of H_2_S gas compounds on the surface of the core shell. The presence of silica as a shell has potentially increased the adsorbent’s stability in several cycles of adsorption-desorption, resulting in low degradation for ZnAc_2_/ZnO/CAC_OS and ZnAc_2_/ZnO/CAC_WS. Thus, it shows a promising performance in capturing H_2_S gas with good stability in the regeneration of adsorbents throughout adsorption-desorption cycles.

## Figures and Tables

**Table 1:** Adsorption capacity of CS/CAC_WS adsorbents.

Adsorbents	Breakthrough Time, T_B_ (min)	Adsorption Capacity, q (mg H_2_S/g)
ZnAc_2_/CAC_WS	8	0.33
ZnAc_2_/ZnO/CAC_WS	13	0.54
ZnAc_2_/TiO_2_/CAC_WS	7	0.29
ZnAc_2_/KOH/CAC_WS	9	0.38

**Table 2:** Adsorption capacity of CS/CAC_OS adsorbents.

Adsorbents	Breakthrough Time, T_B_ (min)	Adsorption Capacity, q (mg H_2_S/g)
ZnAc_2_/CAC_OS	10	0.42
ZnAc_2_/ZnO/CAC_OS	16	0.67
ZnAc_2_/TiO_2_/CAC_OS	13	0.54
ZnAc_2_/KOH/CAC_OS	12	0.50

**Table 3:** Adsorption capacity of CS/CAC_WOS adsorbents.

Adsorbents	Breakthrough Time, T_B_ (min)	Adsorption Capacity, q (mg H_2_S/g)
ZnAc_2_/CAC_WOS	21	0.88
ZnAc_2_/ZnO/CAC_WOS	28	1.17
ZnAc_2_/TiO_2_/CAC_WOS	26	1.09
ZnAc_2_/KOH/CAC_WOS	17	0.71

**Table 4:** Element content on adsorbent surface.

Adsorbents	C	Ca	O	Zn	Ti	K	Si
ZnAc_2_/CAC_WS	40.70	0.73	21.66	11.08	1.14	1.21	23.48
ZnAc_2_/KOH/CAC_WS	36.71	0.32	23.31	7.06	1.33	5.61	25.66
ZnAc_2_/ZnO/CAC_WS	30.96	1.69	28.63	15.98	1.27	1.08	20.39
ZnAc_2_/TiO_2_/CAC_WS	20.51	0.44	27.54	9.43	22.76	1.73	17.59
ZnAc_2_/CAC_OS	43.26	0.45	33.32	12.84	1.53	1.19	7.41
ZnAc_2_/KOH/CAC_OS	38.99	0.48	37.28	9.46	1.09	7.69	5.01
ZnAc_2_/ZnO/CAC_OS	39.78	0.51	38.76	13.3	1.36	1.08	5.21
ZnAc_2_/TiO_2_/CAC_OS	28.33	0.39	34.23	10.11	21.19	1.44	4.31
ZnAc_2_//CAC_WOS	52.19	0.42	32.43	12.16	1.09	1.71	0.00
ZnAc_2_/KOH/CAC_WOS	30.22	1.19	43.50	19.11	1.75	4.23	0.00
ZnAc_2_/ZnO/CAC_WOS	39.06	0.32	45.58	12.35	1.15	1.54	0.00
ZnAc_2_/TiO_2_/CAC_WOS	31.22	0.36	37.70	2.83	26.15	1.74	0.00

**Table 5:** Surface properties of CS/CAC adsorbents.

Adsorbents	BET Surface Area, (m^2^/g)	Pore Volume (cm^3^/g)	Micropore Surface Area, (m^2^/g)	Pore Size (Å)
ZnAc_2_/CAC_WOS (S)	913.24	0.43	757.29	18.75
ZnAc_2_/ZnO/CAC_WOS (S)	939.76	0.45	769.79	19.14
ZnAc_2_/TiO_2_/CAC_WOS (S)	945.02	0.45	775.19	19.09
ZnAc_2_/KOH/CAC_WOS (S)	899.57	0.43	739.48	19.04
ZnAc_2_/CAC_WS (S)	948.93	0.45	758.86	18.89
ZnAc_2_/ZnO/CAC_WS (S)	863.61	0.42	715.08	19.25
ZnAc_2_/TiO_2_/CAC_WS (S)	1006.38	0.47	816.05	18.77
ZnAc_2_/KOH/CAC_WS (S)	932.19	0.44	751.62	19.01
ZnAc_2_/CAC_OS (S)	846.22	0.41	699.19	19.20
ZnAc_2_/ZnO/CAC_OS (S)	913.18	0.44	751.86	19.38
ZnAc_2_/TiO_2_/CAC_OS (S)	999.05	0.48	812.90	18.82
ZnAc_2_/KOH/CAC_OS (S)	865.60	0.39	746.79	18.32
Raw CAC	899.05	0.42	730.02	18.82

**Table 6:** Thermal analysis of CS/CAC adsorbents.

Adsorbents	Temperature Range (°C)	Wt. Loss (%)
ZnAc_2_/CAC_WOS	25–100	8.0
	100–400	1.9
	400–600	2.5
ZnAc_2_/ZnO/CAC_WOS	25–100	7.5
	100–400	2.8
	400–600	2.4
ZnAc_2_/TiO_2_/CAC_WOS	25–100	10.8
	100–400	2.3
	400–600	2.2
ZnAc_2_/KOH/CAC_WOS	25–100	10.3
	100–400	2.6
	400–600	2.5
ZnAc_2_/ZnO/CAC_WS	25–100	4.9
	100–400	2.5
	400–600	2.3
ZnAc_2_/ZnO/CAC_OS	25–100	18.6
	100–400	2.5
	400–600	2.0
ZnAc_2_/CAC	25–100	6.9
	100–400	6.1
	400–600	3.1
Raw CAC	25–100	15.4
	100–400	4.8
	400–600	4.9

## Data Availability

Not applicable.
